# Bacteria-Polymer Composite Material for Glycerol Valorization

**DOI:** 10.3390/polym15112514

**Published:** 2023-05-30

**Authors:** Magdalena Ripoll, Nicolás Soriano, Sofía Ibarburu, Malena Dalies, Ana Paula Mulet, Lorena Betancor

**Affiliations:** 1 Department of Biotechnology, Universidad ORT Uruguay, Mercedes 1237, Montevideo 11100, Uruguay; ripoll_m@ort.edu.uy (M.R.); ns222430@fi365.ort.edu.uy (N.S.); ibarburu@ort.edu.uy (S.I.); md209091@fi365.ort.edu.uy (M.D.); mulet@ort.edu.uy (A.P.M.); 2 Graduate Program in Chemistry, Facultad de Química, Universidad de la República, Av. Gral. Flores 2124, Montevideo 11800, Uruguay

**Keywords:** hybrid polymers, biocatalysis, bacterial immobilization, nanomaterials, glycerol, *Gluconobacter*

## Abstract

Bacterial immobilization is regarded as an enabling technology to improve the stability and reusability of biocatalysts. Natural polymers are often used as immobilization matrices but present certain drawbacks, such as biocatalyst leakage and loss of physical integrity upon utilization in bioprocesses. Herein, we prepared a hybrid polymeric matrix that included silica nanoparticles for the unprecedented immobilization of the industrially relevant *Gluconobacter frateurii* (Gfr). This biocatalyst can valorize glycerol, an abundant by-product of the biodiesel industry, into glyceric acid (GA) and dihydroxyacetone (DHA). Different concentrations of siliceous nanosized materials, such as biomimetic Si nanoparticles (SiNps) and montmorillonite (MT), were added to alginate. These hybrid materials were significantly more resistant by texture analysis and presented a more compact structure as seen by scanning electron microscopy. The preparation including 4% alginate with 4% SiNps proved to be the most resistant material, with a homogeneous distribution of the biocatalyst in the beads as seen by confocal microscopy using a fluorescent mutant of Gfr. It produced the highest amounts of GA and DHA and could be reused for up to eight consecutive 24 h reactions with no loss of physical integrity and negligible bacterial leakage. Overall, our results indicate a new approach to generating biocatalysts using hybrid biopolymer supports.

## 1. Introduction

Biotechnology offers a greener panorama in future product and material manufacturing as the industry is transitioning toward net-zero carbon processes [1]. In general, biotransformations necessitate improvements and optimization studies for feasible and effective applications. In bacterial biotransformations, the attachment of bacteria to a carrier material, known as immobilization, can improve productivity and production costs through stabilization of the catalyst, repeated utilization, and ease of separation [2,3,4]. Moreover, biotransformations catalyzed by immobilized bacteria are often carried out in cleaner backgrounds, simplifying downstream processing units [5,6,7].

An ideal immobilization matrix should possess physical durability, stability, hydrophilicity, inertness, easy functionalization, biocompatibility, resistance to microbial attack, and cost-effectiveness. Various organic and inorganic matrices are available for bacterial immobilization [8]. The inorganic matrices include sintered glass, ceramics, carbon-based materials, diatomite, and quartz, while the natural matrices include collagen, agar, agarose, cellulose, chitosan, and alginate, and synthetic matrices consist of polymers, such as acrylamide, polyurethane, and polyvinyl alcohol [9]. Both inorganic and organic materials may be used following different strategies for bacterial immobilization, such as coupling to solid surfaces, encapsulation, aggregation, and entrapment, which are frequently used. Coupling involves attaching cells to a surface naturally or by adding binding agents. Encapsulation entails confining bacteria, where they are separated from the reaction medium by a barrier. Aggregation involves the formation of cell aggregates linked naturally or by the addition of flocculating or binding agents [10].

A thoughtful choice of the methodological approach and materials is crucial for the creation of efficient immobilized biocatalysts [11,12,13]. In terms of the materials used as supports, the selection must consider bioprocess conditions, such as pH, temperature, agitation, or additives, as they may affect said materials [14]. Additionally, the biocatalyst may be influenced by the nature of the immobilization matrix as it may change its biological properties [15]. Moreover, physical parameters, such as pore size or total surface area, directly impact the amount of biocatalyst immobilized as well as the partition of substrates and products affecting the productivity of biotransformations. Hence, the study of new supports for biocatalyst immobilization aiming for more efficient bioconversions is not only timely but also necessary to perfect bioprocesses for their industrial implementation.

Natural polymers such as agar, agarose, chitosan, cellulose, collagen, carrageenan, and alginate are non-toxic, biocompatible, and biodegradable, which brings them closer to the concept of ideal carriers or immobilization matrixes for biocatalysts [16]. They contribute to the sustainability of bioprocesses over synthetic non-biodegradable polymers, such as polystyrene, or inorganic processed materials, such as sintered glass. However, it is frequent that upon bacterial immobilization, biocatalytic activity decreases either by loss of activity or due to partition problems of the substrates and products [17]. Moreover, these polymers may degrade or present structural issues after sustained or repeated use, causing matrix rupture and bacterial leakage to the reaction medium. These problems might be alleviated by varying the physical properties of the immobilization material.

Recently, the use of combined or polymeric hybrid materials for the integration of biocatalysts has opened a myriad of possibilities in which each of the materials adds advantages to the final heterogeneous biocatalyst [18,19,20,21]. In particular, the integration of nanomaterials and polymers for bacterial immobilization, although scarcely reported so far, has very recently been demonstrated to improve the physical properties of the composite for specific applications [22].

In this work, we have investigated the preparation of hybrid polymeric materials of alginate and siliceous nanoparticles and their impact on the immobilization of a strain of *Gluconobacter.* This type of bacteria has industrial relevance as it catalyzes the incomplete oxidation of sugars and alcohols, generating products of chemical, pharmaceutical, and cosmetic interest [5,23,24,25,26]. To test the different support materials, we have selected the type strain *Gluconobacter frateurii* NBRC103465 (Gfr) [27] that transforms and upgrades glycerol into dihydroxyacetone (DHA) and glyceric acid (GA) [23]. The biotransformation has industrial and environmental relevance, as glycerol is inevitably generated as a by-product of the biodiesel industry [28], and its valorization may help diminish the environmental problems associated with its disposal in line with the economics of the biofuel industry and the biorefinery concept. A cost-effective and sustainable process is a must in this transformation. It competes with the easier but contaminated waste disposal via incineration of the substrate [29], the high yield but multi-step biotechnological production of DHA with bacteria in the growing phase [30], and the chemical synthesis of GA, which presents low selectivity and requires heavy metal catalysts as well as extreme reaction conditions [31,32]. We, therefore, worked under the hypothesis that the use of a polymeric hybrid material would improve the properties of an immobilized biocatalyst of Gfr for a more sustainable biotransformation of glycerol into DHA and GA.

## 2. Materials and Methods

### 2.1. Materials

Glucose, K_2_HPO_4_, NaH_2_PO_4_, CaCl_2_.2H_2_O, HCl, and pure glycerol (Carlo Erba Reagents, Val-de-Reuil, France). Peptone (PanReac AppliChem, Barcelona, Spain). Yeast extract and agar (Oxoid, Basingstoke, UK). MgSO_4_.7H_2_O (J.T. Baker, PA, USA). KH_2_PO_4_ (Macron Chemicals, PA, USA). Tetramethyl orthosilicate (TMOS) (Merck, Darmstadt, Germany). Agarose (Bioron, Römerberg, Germany). Alginate (Sigma, Burlington, VT, USA). Montmorillonite (MT) (Aldrich, Burlington, VT, USA).

### 2.2. Bacterial Strains and Plasmids

*Gluconobacter frateurii* NBRC103465 (Gfr) was obtained from the National Institute of Technology and Evaluation (NITE) (Tokyo, Japan).

Plasmid MYMH 464 was a gift from Meng How Tan (Addgene plasmid #138068; http://n2t.net/addgene:138068; RRID:Addgene_138068). Plasmid pSEVA231-CRISPR was a gift from Víctor de Lorenzo (Addgene plasmid #138712; http://n2t.net/addgene:138712; RRID:Addgene_138712)

### 2.3. Plasmid Construction and Bacterial Modification

The p104-mCherry cassette was obtained from the MYMY464 plasmid and cloned into the pSEVA231-CRISPR vector using XbaI and BamHI enzymes. The construction was amplified in *E. coli* DH5α and named p104–pSEVA231–mCherry. The fluorescent strain *G. oxydans* p104–pSEVA231–mCherry was generated by electroporation of the p104–pSEVA231–mCherry plasmid into electrocompetent *G. oxydans* cells. Positive clones were selected with 50 µg/mL kanamycin.

### 2.4. Bacterial Inoculum Preparation

The Gfr precultures were prepared as described by Habe et al. [33] in 3 mL of glucose-containing medium (5 g/L peptone, 5 g/L yeast extract, D-glucose 5 g/L, MgSO_4_.7H_2_O 1 g/L, pH 6.5) and were incubated at 30 °C and 180 rpm for 16 h. Cultures of 250 mL were inoculated with 6 mL of Gfr preculture and incubated at 30 °C and 180 rpm in 1 L flasks containing growth medium with pure glycerol (glycerol 100 g/L, peptone 9 g/L, yeast extract 1 g/L, KH_2_PO_4_ 0.9 g/L, K_2_HPO_4_ 0.1 g/L, MgSO_4_.7H_2_O 1 g/L, pH 6.0) [33]. At an OD_600 nm_ value of 1, a volume corresponding to 20 mg dry cell weight (DCW) was centrifuged for 15 min at 5000 rpm and 4 °C. The bacterial pellet was washed with 30 mM phosphate buffer (NaH_2_PO_4_ 4.14 g/L, K_2_HPO_4_ 5.23 g/L, pH 7.0), hereinafter referred to as washing buffer, to remove debris from the growth medium and finally centrifuged at 5000 rpm at 4 °C for 15 min again, discarding the supernatant. The bacterial pellets were stored at −20 °C until later use.

### 2.5. Silica Nanoparticles (SiNps) Synthesis

Silica nanoparticles were synthesized following a protocol described in previous work [34]. Briefly, a mixture of 20 mL of sodium phosphate buffer 100 mM pH 8.0, 5 mL of a 5% solution of polyethyleneimine (MW 1300), and 5 mL of previously hydrolyzed tetramethyl orthosilicate (TMOS) was prepared in a 50 mL tube. The hydrolyzation of TMOS was performed by adding 942 µL of TMOS to 6 mL of HCl 1 mM and subsequently mixing the resulting solution using a vortex. The SiNps were immediately formed upon the addition of the hydrolyzed TMOS. To remove any remaining reactants present in the buffer, the SiNps suspension was centrifuged at 5000 rpm for 10 min and washed thrice with 30 mL of distilled water.

### 2.6. Bacterial Immobilization

For the preparation of the agar and agarose beads, 20 mg DCW (5 × 10^10^ UFC) was mixed with 3 mL of a 3% agar or agarose solution [35]. The homogeneous mixture was added dropwise to a beaker containing cold sunflower oil while stirring. The resulting beads (AR3 and AE3 for agar and agarose, respectively) were separated using a strainer and washed with hexane and washing buffer. For the preparation of the alginate beads, 20 mg DCW was mixed with 2 mL of 6% alginate and 1 mL of water (A4), 1 mL of montmorillonite (MT) at 30 or 120 mg/mL (A4M1 and A4M4), or SiNps (A4S1 and A4S4) at 30 or 120 mg/mL for a final alginate concentration of 4%. The alginate or hybrid mixtures containing the cells were added dropwise using a syringe pump (20 µL/s) to 30 mL of CaCl_2_ 50 mM. The beads were incubated for 1 h at 4 °C, separated using a strainer, and washed thoroughly with distilled water.

The dimensions of all the immobilized preparations were measured using ImageJ software (V 1.53e) (National Institutes of Health, Bethesda, MD, USA).

### 2.7. Scanning Electron Microscopy Analysis

The alginate bead morphology was studied using scanning electron microscopy (SEM). The beads were frozen, freeze-fractured in liquid nitrogen, and gold coated, as described by Simoni et al. [36]. The ice was removed from the fractured specimens by vacuum sublimation (freeze-drying) using a LyoQuest lyophilizer (Telstar, Barcelona, Spain. The samples were then visualized by high-resolution field emission environmental scanning electron microscopy using a Quanta FEG 250 microscope (FEI, OR, USA) under low vacuum conditions with a beam voltage of 10 kV and a magnification of 100× or 1600×.

### 2.8. Texture Analysis

The analyses were carried out using a TA.XT.Plus texture analyzer from Stable Micro Systems. A total of 10 beads for each condition (4% alginate (A4), 4% alginate + 1% MT (A4M1), 4% alginate + 4% MT (A4M4), 4% alginate + 1% SiNps (A4S1), or 4% alginate + 4% SiNps (A4S4)) was tested. The probe was a cylindric P2, and the cell load was 5 kg. The test mode was compression, the pre-test speed was 5 mm/s, the test speed was 1 mm/s, and the post-test speed was 5 mm/s. The target mode was set to Strain (100%). The trigger type was set as Auto (Force), and the trigger force was 1 N. The break mode was set to Level and the break sensitivity was 10 g.

Statistical analyses of variance (ANOVA) were performed using Prism 8.0.1 software (GraphPad Software, San Diego, CA, USA).

### 2.9. Confocal Microscopy Analysis

The immobilized biocatalyst was analyzed by confocal microscopy using a confocal ZEISS LSM 800. Hybrid and alginate polymeric matrices containing the fluorescent strain *G. frateurii* p104–pSEVA231–mCherry were whole-mounted in distilled water. A × 63 (1.3 numerical aperture) oil-immersion objective was used to acquire Z-stacks, and maximum intensity projections of the optical sections were created with ImageJ software (V 1.53e).

### 2.10. Conversion of Glycerol to GA and DHA

Pure glycerol conversions with resting cells in shake flasks were carried out as described in previous work using starting glycerol concentrations of 200 g/L in water [23]. Glycerol conversions with immobilized preparations were carried out in 250 mL Erlenmeyer flasks containing 30 mL of 200 g/L glycerol in water. The inoculum of each reaction was 20 mg DCW immobilized in 3 mL of 3% agar or agarose (AR3 and AE3, respectively), 3 mL of 4% alginate (A4), 4% alginate + MT 1% (A4M1), 4% alginate + MT 4% (A4M4), 4% alginate + SiNps 1% (A4S1), or 4% alginate + SiNps 4% (A4S4). Samples were periodically withdrawn from the reactions and analyzed by HPLC. The turbidity of the reaction medium was determined by spectrophotometry at 600 nm using a Shimadzu UV-1800 spectrophotometer (Kyoto, Japan).

### 2.11. Reuse of Resting and Immobilized Cells

Resting as well as immobilized cells were reused in 24 h reactions. Each time, the cells and beads containing the immobilized cells were harvested from the reaction flasks using either centrifugation or a strainer in the case of the beads and washed once with 10 mL of distilled water. After the washing, the biocatalysts were placed in a 250 mL flask containing 30 mL of 200 g/L of glycerol in water for another round of reaction.

The residual activity was calculated after each use considering the obtained yield (g/L) of use 1 as 100%.

### 2.12. HPLC Analysis

During all the experiments, 400 µL samples were taken periodically. The samples were centrifuged at 15,000 rpm for 15 min at 4 °C. Each sample was subsequently filtered with a 0.22 μm filter treated with 2% polyvinylpyrrolidone (PVP) and analyzed by HPLC. The production of GA and DHA was analyzed using Shimadzu Nexera X2 HPLC equipment (Kyoto, Japan) with a diode array detector. The column was an Aminex^®^ HPX-87C 300 × 7.8 mm (Bio-Rad, Hercules, CA, USA), with a pre-column loaded with a 4 × 3.0 mm SecurityGuard™ cartridge from Phenomenex Carbo-H columns (Phenomenex, Torrance, CA, USA). The column was incubated at 70 °C, and the mobile phase was 5 mM sulfuric acid. Detection was carried out at 210 nm for GA, 271 nm for DHA, and 190 nm for glycerol, with a 0.6 mL/min flow rate for 19 min. The recorded retention times were GA (12.9 min), DHA (16.7 min), and glycerol (14.7 min). The samples were injected using a volume of 20 μL. Calibration curves were constructed for GA (0.02–33 g/L), DHA (0.15–5 g/L), and glycerol (0.15–20 g/L). The samples were analyzed using LabSolutions software (V5.111) (Shimadzu, Kyoto, Japan).

The data presented throughout the manuscript reflect the mean values of three independent experiments. The error bars represent the standard deviation.

## 3. Results and Discussion

Agar, agarose, and alginate are polymers commonly used as matrices in bacterial immobilization [35,37]. Their selection of inorganic or synthetic polymers is often based on the fact that they are affordable and chemically inert. Moreover, the obtention of immobilized biocatalysts with these polymers is achieved by a facile entrapment of bacteria upon mixing the biomass with an aqueous agar, agarose, or alginate solution and subsequent dropwise addition of the resulting mixture into a solution of CaCl_2_ (for alginate) or cold sunflower oil (for agar and agarose).

It is worth noting that the CaCl_2_ concentration may affect the alginate properties among other variables [38] (i.e., alginate concentrations, gelation temperatures, incubation times, etc.) that were fixed in this study in order to focus on the advantages of the combination of different materials.

In a previous study, we reported the unprecedented immobilization of *Gluconobacter oxydans* (Gox) in 3% agar (AR3) and 3% agarose (AE3). Gox is also able to catalyze the conversion of glycerol, but only to DHA. The work established that they were suitable matrices for DHA production and allowed the biocatalyst’s repeated use in water, which was proven unfeasible with resting cells [5].

Herein, we evaluated AR3, AE3, and 4% alginate (A4) previous to the preparation of hybrid polymers for the immobilization of a different strain of *Gluconobacter*, Gfr, and its potential use in the preparation of not only DHA but also GA in the glycerol transformation. Despite alginate, agar, and agarose being well-known matrices for bacterial immobilization, to the best of our knowledge, there are no previous reports of them being used for Gfr immobilization and/or the production of GA. Therefore, it was necessary to evaluate the immobilization of this strain in the natural polymers by themselves.

Following the standard approach for entrapment in the different polymers described in the Methods, we obtained three different immobilized biocatalysts from 20 mg of dried cell weight. The number of beads obtained as well as the resulting beads’ mean diameter and size and shape distribution varied within the matrixes and immobilization techniques (Figure S1, Table S1). These immobilized preparations were then tested in bioconversion reactions.

**Figure 1:**
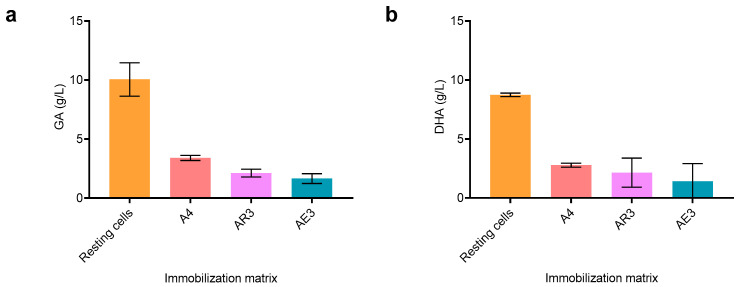
Glycerol conversion with *Gluconobacter frateurii* (Gfr) immobilized in different matrixes (**a**) Glyceric acid (GA) production in 20 h. (**b**) Dihydroxyacetone (DHA) production in 20 h. Reactions were performed in 30 mL of 200 g/L glycerol in water using 20 mg Dry Cell Weight (DCW) immobilized in the different supports.

In a previous study, we established that elevated glycerol concentrations produce higher GA yields [23]. In accordance with those results, we selected a concentration of 200 g/L of glycerol to carry out the biotransformation with the immobilized preparations. All the immobilized preparations were able to produce GA from 200 g/L of glycerol in water, although the conversions were lower than that obtained with resting cells (Figure 1). A decrease in product formation after immobilization is commonplace, as there may be partition issues that can affect substrate and product diffusion to and from the beads. However, immobilized biocatalysts are often protected against inactivating agents that can be present in a feedstock, such as crude glycerol. Therefore, further studies are necessary to evaluate the contribution of this technology to the process. Additionally, immobilization enables easier reusability and facile separation, which is a desirable characteristic in a biocatalyst with potential industrial application.

After 20 h of reaction, GA production was slightly superior to A4 preparations, while DHA production was comparable between all the preparations (Figure 1). The standard deviation obtained in the production of DHA for agar and agarose seems significantly higher than that of the GA concentration. Given that these products are formed by different membrane-associated enzymes, glycerol dehydrogenase (GDH) for DHA and alcohol dehydrogenase (mADH) for GA, it is plausible that slight differences in the temperature during the immobilization process could have impacted GDH residual activity, thus producing beads with varying DHA producing abilities.

Altogether, the A4 preparations performed better than AR4 and AE4. Thus, this immobilization technique was selected for further experiments.

**Scheme 1:**
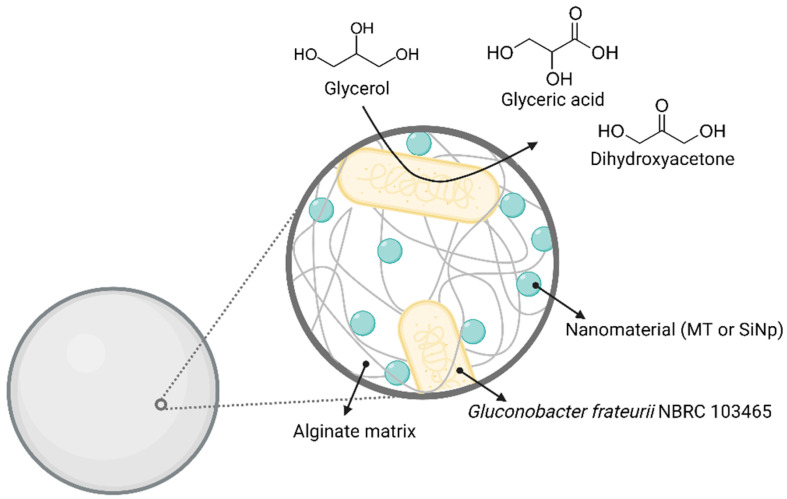
Schematic representation of hybrid immobilized preparations combining alginate and nanomaterials. MT: montmorillonite, SiNp: Silica nanoparticle.

As mentioned before, oftentimes, natural polymer matrices are labile, making their sustained reuse difficult and causing bacterial leakage from the matrix to the medium. In fact, in a recent work, we observed that alginate preparations of *G. oxydans* lost their complete structural integrity after four repeated uses [39]. An interesting alternative to enhance the structural properties of hydrogels is the addition of nanostructured materials to the matrix. Examples of these nanomaterials are silica nanoparticles (SiNps) and clays, such as montmorillonite (MT) [40,41,42,43,44]. Through their addition to the alginate matrix, the mechanical resistance can be improved, avoiding matrix breakage. In addition, the incorporation of these silicious nanomaterials could contribute to a decrease in bacterial leakage through the formation of electrostatic interactions. Indeed, bacteria are known to adhere to the surface of MT [45,46,47], while the properties of biomimetic silica, such as a superficial charge, may contribute to bacterial adhesion [34,48]. It is worth noting that both materials are inherently green. MT is a natural clay, and the SiNps to be used are biomimetic, that is, synthesized without the need for an organic solvent and heat, and they are smart, as they can disintegrate upon usage [49]. We, therefore, combined alginate and these two nanomaterials to immobilize Gfr to improve the matrix’s properties (Scheme 1).

**Figure 2:**
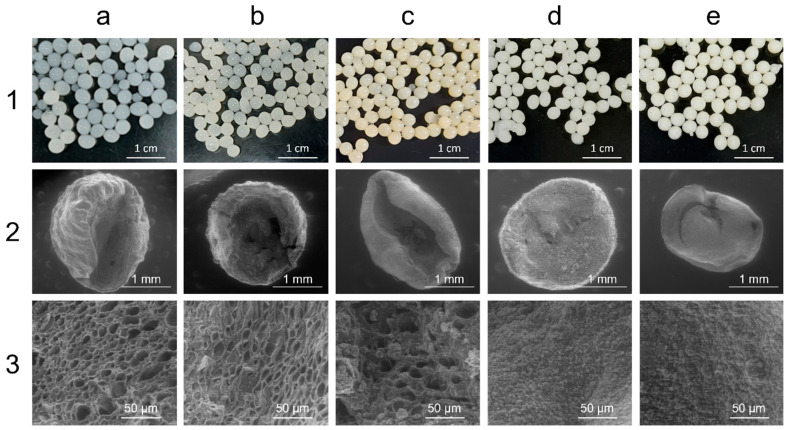
Gfr immobilization on hybrid materials. (**1**) Macroscopic appearance of (**a**) A4. (**b**) A4M1. (**c**) A4M4. (**d**) A4S1. (**e**) A4S4. (**2**) Scanning electron microscopy image (100×) of (**a**) A4. (**b**) A4M1. (**c**) A4M4. (**d**) A4S1. (**e**) A4S4. (**3**) Scanning electron microscopy image (1600×) of (**a**) A4. (**b**) A4M1. (**c**) A4M4. (**d**) A4S1. (**e**) A4S4.

We evaluated the addition of SiNps and MT in two different concentrations (1% and 4% *w*/*v*), using alginate-only beads (A4) as a control. The immobilization protocol was the same as the one used for the A4 immobilized preparations, but with an initial step in which the bacterial pellets were mixed with the nanomaterials. The resulting preparations were named as follows: 4% alginate with 1% MT (A4M1), 4% alginate with 4% MT (A4M4), 4% alginate with 1% SiNps (A4S1), and 4% alginate with 4% SiNps (A4S4). All hybrid preparations presented similar sizes (3.6 ± 0.4 mm on average) and spherical shapes, with slight color differences associated with the addition of the nanomaterials (Figure 2). 

Further characterization of the hybrid preparations was carried out by scanning electron microscopy (SEM). To prevent significant structural collapse, each bead was initially frozen and freeze-fractured using liquid nitrogen and subsequently dried by lyophilization, as described by Simoni et al. [36]. It is clear from the SEM analysis that the internal structure of the immobilized preparations changed upon the addition of the siliceous materials (Figure 2). Moreover, the micrographs showed that A4S1 and A4S4 were the preparations that collapsed the least (Figure 2, panels d2 and e2, respectively), while the other preparations showed clear signs of structural collapse (Figure 2, panels a2, b2, and c2). This indicates that the matrices containing SiNps are more rigid and might be more resistant, which is beneficial for bioconversion purposes. This observation may be linked to an increased number of hydrogen bonds between the two materials, as proposed by Yang et al. [50], who studied composite aerogels made of agarose and SiO_2_ for thermal insulation applications. Additionally, the integration of agarose and alginate beads with Si materials could also be driven by polar interactions between the uncharged siloxane groups and the hydroxyl groups of the alginate biopolymer [51].

Close-ups of the cross-sections of the resulting beads revealed differences in the porosity of the matrices. Larger pores can be observed in the control A4 bead (Figure 2, panel a3), while the incorporation of MT resulted in less porosity, as clay particles can be seen clogging the pores. This decrease in porosity seems to be concentration-dependent, evidenced by the differences observed in the micrographs (Figure 2, panels b3 and c3). Similar results were obtained by Etcheverry et al. [51] when investigating agar and montmorillonite materials for the removal of pollutants and by Polat et al. in wound dressing applications [52]. Furthermore, the immobilized preparations that contained SiNps (A4S1 and A4S4) presented a distinctive morphology as no evident pores were seen in either of the micrographs (Figure 2, panels d3 and e3). A tighter matrix mesh can be correlated with greater mechanical resistance and may allow for a decrease in bacterial leakage, a common problem regarding alginate matrices [39].

**Figure 3:**
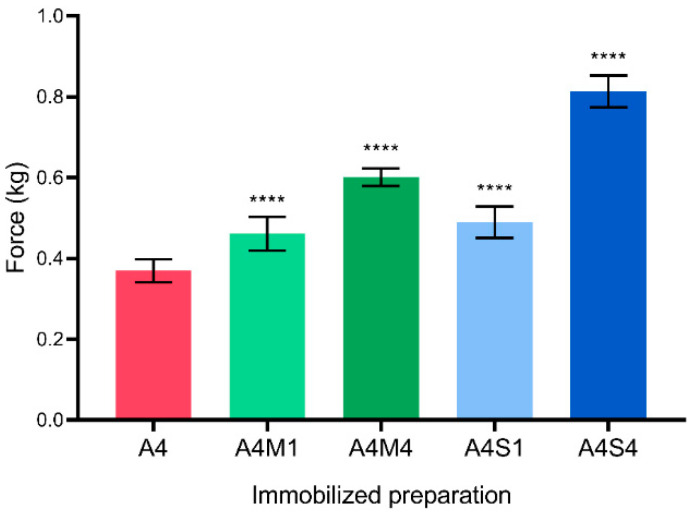
Resistance analysis of immobilized preparations performed using a texture analyzer. **** = *p*-value < 0.0001.

Knowledge of the properties of polymers applied to the field of bioengineering is fundamental, as their physical properties have proven in the past to play a role in the mass transfer that occurs through the beads in response to external forces, their mechanical resistance, etc. We, therefore, proceeded to further characterize the mechanical properties of the hybrid alginate biocatalysts. All the preparations were assessed for their mechanical resistance. The force needed to break the preparations was evaluated using a texture analyzer (Figure 3).

In all the cases, the addition of the nanostructured materials resulted in significantly more resistant hybrid preparations in comparison to the control (A4). The higher the percentage of MT and SiNps, the greater the resistance to rupture, and A4S4 was the immobilized preparation that presented the best results.

To evaluate the ability of the hybrid preparations to synthesize GA and DHA, glycerol conversion reactions were carried out at an initial substrate concentration of 200 g/L (Figure S2, Table 1). The initial velocities of the product formation differed amongst the immobilized preparations, especially in GA production with A4S1 and A4S4. Nevertheless, after 170 h, GA production was similar for every immobilized preparation, including the control (A4), reaching concentrations of about 8 g/L. DHA production, however, was better with the hybrid preparations including SiNps, as 9.7 ± 0.3 g/L and 10.8 ± 0.9 g/L were obtained with the A4S1 and A4S4 preparations, respectively. This could be explained by better resistance of the GDH enzyme (responsible for glycerol oxidation into DHA) to this immobilization matrix or by lesser diffusional restrictions imposed on the DHA by this material. It is noteworthy that after 24 h of reaction, the A4S1 and A4S4 preparations started presenting a yellow hue, which further intensified throughout the duration of the experiment and seems to be dependent on the SiNps concentration. Control experiments were carried out for 24 h in the absence of Gfr and the absence of glycerol with no change in color (Figure S3). This indicates that glycerol oxidation by Gfr caused the change in coloration, probably by promoting polyethyleneimine’s amino group’s oxidation remaining from the SiNps synthesis.

An evaluation by UV-VIS spectrophotometry of the reaction supernatants after 170 h of production showed that all the hybrid preparations delivered the cleanest reaction crudes (Figure S4, Table 1). On the contrary, the A4 preparations showed higher turbidity, probably due to matrix damage and bacterial leakage. These results are encouraging, as a cleaner reaction supernatant requires less downstream processing.

Given the better results obtained with the A4 immobilized preparations, an experiment was conducted to determine if the addition of SiNps to the alginate matrix contributes to the decrease in bacterial leakage from the immobilized preparations to the reaction medium. For this experiment, a glycerol conversion reaction was simulated, but the reaction medium was replaced with sterile water. In contrast to previous experiments, Gfr was immobilized in aseptic conditions in the A4 and A4S4 matrices. All the reagents and materials were sterilized before use. Samples of the reaction supernatants were taken after 24 h and plated, and the colony-forming units per mL (CFU/mL) obtained in each case were counted. The incorporation of SiNps into the A4S4 preparations contributed to diminishing bacterial leakage, as the UFC/mL detected in the hybrid supernatant (A4S4) was merely 10% of the total UFC/mL detected in the control supernatant (A4) (150 ± 71 UFC/mL and 1310 ± 297 UFC/mL, respectively). A plausible explanation for this phenomenon may be related to the establishment of ionic interactions between Gfr and the matrix. SiNps are positively charged due to polyethyleneimine, while the lipopolysaccharides present in the outer membrane of Gram-negative bacteria, such as Gfr, have a negative charge [34,48]. Moreover, the better physical resistance of this material linked to the tighter matrix mesh, as seen by SEM analysis, could be related to reduced pore sizes that diminish bacterial leakage. It is noteworthy that bacterial leakage from both preparations was low, as 20 mg of dry cell weight of bacteria equals approximately 5 × 10^10^ UFC.

**Figure 4:**
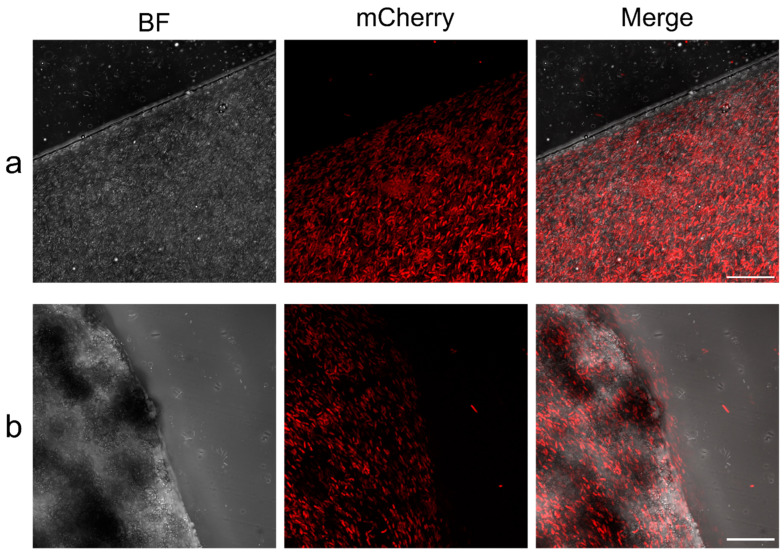
Confocal analysis of immobilized preparation of a fluorescent Gox within (**a**) A4 and (**b**) A4S4. BF: Brightfield, mCherry: mCherry fluorescent protein. Scale bar = 20 µm.

To study whether the bacteria were integrated into the hybrid and non-hybrid polymer, both the A4 and A4S4 preparations were analyzed by confocal microscopy. For this experiment, a mutant of *Gluconobacter oxydans* (Gox) expressing mCherry fluorescent protein was used in the same immobilization experiments developed for Gfr. Both catalysts showed a similar homogeneous bacterial integration on the material. However, the images presented slight differences that seem to be related to the hardness of the catalysts, as a cleaner cut was achieved in the A4 preparations in comparison to that obtained in the A4S4 preparations (Figure 4). This similar bacterial distribution is consistent with the comparable DHA and GA conversion results that were previously demonstrated.

**Figure 5:**
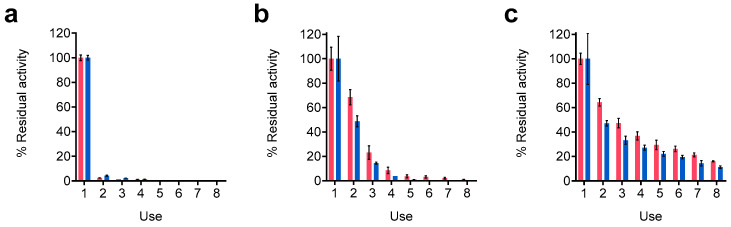
Reusability evaluation of Gfr-based biocatalysts. (**a**) Repeated conversion of pure glycerol using resting cells. (**b**) Repeated conversion of pure glycerol using A4 immobilized preparations. (**c**) Repeated conversion of pure glycerol using A4S4 immobilized preparations. Residual GA production activity (red), residual DHA production activity (blue). Each use lasted 24 h.

Multiple or repeated batch transformation cycles are a paramount goal when designing immobilized biocatalysts for bioconversions. The possibility of reusing the same biocatalysts reduces costs, intensifying and increasing the sustainability of the process. One determinant property of the immobilized biocatalysts for this purpose is the mechanical resistance of the immobilization matrix, which while maintaining its physical integrity may protect the biological catalyst for longer periods from harsh conditions and its leakage to the reaction medium. We, therefore, studied the reusability of the A4S4 preparations in comparison with resting cells by evaluating the residual activity after 24 h glycerol conversion reactions (Figure 5). For each use, a comparison of the analysis of the substrates and products for t_0_ and t_24_ h was conducted. The resting cells of Gfr could not be reused successfully, as both GA and DHA production diminished almost completely after the first use (Figure 5a). Regarding the immobilized preparations, a control was carried out with the A4 preparations. As previously mentioned, alginate matrices can sometimes be labile, hindering their reusability. In line with that, repeated glycerol conversion control reactions with the A4 preparations showed a sensible loss in both residual activity and structure. Residual GA production activity diminished to 23.1 ± 5.6% after the third consecutive use and continued under 20% for the rest of the experiment (Figure 5b). Similar results were obtained for DHA, as its production diminished to 14.5 ± 0.9% after three uses and ceased completely after five uses. Moreover, the structural integrity of the A4 preparations was compromised after five consecutive uses, when the beads started to show clear signs of swelling and physical disruption. After eight uses, all the beads were damaged (Figure S5). In contrast, the A4S4 preparations were successfully reused in eight successive glycerol 24 h conversion reactions (Figure 5c). The reusability of the A4S4 preparations was vastly superior to that of the A4 preparations. The residual activity was maintained above 20% for GA in seven subsequent uses, slightly diminishing to 16.0 ± 0.5% after eight subsequent uses. The DHA residual activity was also maintained above 20% for six uses and diminished to 11.0 ± 1.0% after eight subsequent uses. Unlike the A4 preparations, there were no signs of swelling or damage to the immobilization matrix after eight consecutive uses. Remarkably, the average bead diameter for the A4S4 preparations descended from 3.9 ± 0.3 mm to 2.6 ± 0.3 mm (Table S2).

## 4. Conclusions

The industrial application of immobilized biocatalysts significantly depends on the proper design of the immobilization strategy and the material used as support. High activity and reusability are both desired properties when immobilizing biocatalysts. Biopolymers have several advantages as support for bacterial integration but often lack physical resistance, which ultimately impacts bioprocess productivity. Herein, we have demonstrated that several organic matrices allow the immobilization of Gfr, an efficient biocatalyst that can valorize glycerol into GA and DHA and which had not been heterogenized before nor used immobilized in the studied biotransformation. Moreover, in aiming to improve the biocatalyst properties, we have proven that the incorporation of nanosized siliceous materials into alginate immobilized preparations of Gfr enables better reusability, improved physical resistance, and significantly diminishes bacterial leakage. The immobilized preparation that combined alginate 4% with SiNps 4% (A4S4) was successfully used for glycerol conversion. The strategy described here provides a simple and effective approach for biotechnologists to create stable, solid-phase biocatalysts, not only for glycerol valorization but also for other bioconversions that seek to improve their sustainability. Moreover, we envision that the strategy developed here of integrating Si nanoparticles into alginate for biocatalyst immobilization could be extended to other natural polymer applications, such as tissue engineering, drug release systems, or a bioartificial medium for cell culture.

## Figures and Tables

**Table 1:** GA and DHA production from glycerol with various alginate-based immobilized preparations of Gfr.

Immobilized Preparation	Initial Bead Diameter (mm)	Final Bead Diameter (mm) ^a^	Maximum GA Yield (g/L) ^a^	Maximum DHA Yield (g/L) ^a^	Final DO_600 nm_ Value
A4	3.7 ± 0.5	3.5 ± 0.5	7.7 ± 0.5	4.8 ± 0.2	0.368 ± 0.042
A4M1	3.7 ± 0.3	3.5 ± 0.3	8.3 ± 0.5	6.1 ± 0.5	0.095 ± 0.021
A4M4	3.5 ± 0.4	3.1 ± 0.4	8.1 ± 0.6	5.6 ± 0.1	0.106 ± 0.031
A4S1	3.5 ± 0.3	2.8 ± 0.3	8.9 ± 0.2	9.7 ± 0.3	0.137 ± 0.154
A4S4	3.9 ± 0.4	2.7 ± 0.3	8.7 ± 0.8	10.8 ± 0.9	0.039 ± 0.007

^a^ After 170 h.

## Data Availability

Raw data is available upon request.

## Supplementary Materials

The following supporting information can be downloaded at https://www.mdpi.com/article/10.3390/polym15112514/s1. Figure S1: Immobilized preparations of Gfr using different matrices; Figure S2: Glycerol bioconversions with Gfr immobilized preparations; Figure S3: Evaluation of color change after 24 h in A4S4 preparations; Figure S4: Reaction supernatants after 170 h of glycerol conversion reactions carried out by different Gfr immobilized preparations; Table S1: Immobilization of Gfr in natural polymers; Table S2: Diameter evaluation of A4 and A4S4 preparations before and after eight consecutive uses.
