# Supplementary material for: Bacteria-Polymer Composite Material for Glycerol Valorization
Source: Polymers (Basel). 2023 May 30;15(11):2514. doi: 10.3390/polym15112514 (PMC10255872; doi:10.3390/polym15112514)
Supplement: Supplementary file 1 [file polymers-15-02514-s001.zip › polymers-2325175-SI.pdf]

Supplementary Materials

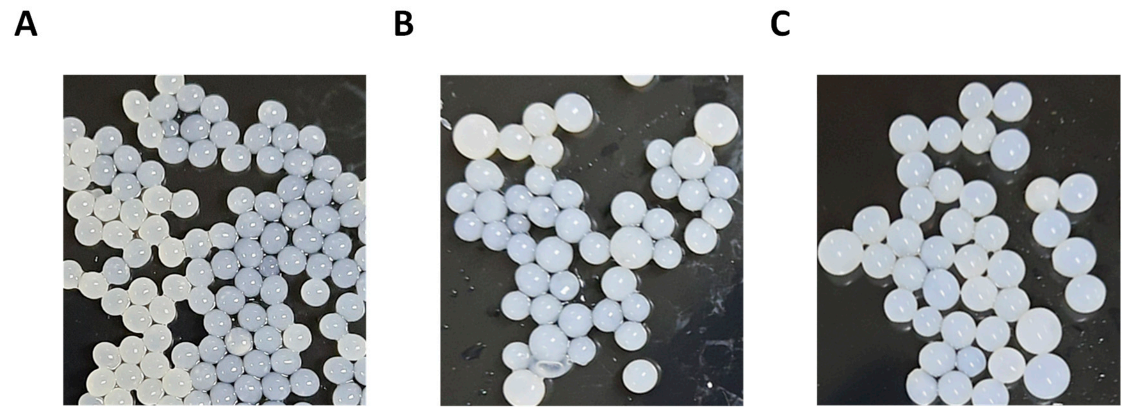

Figure S1. Gfr immobilized preparations using different matrixes. A) A4. B) AR3. C) AE3.

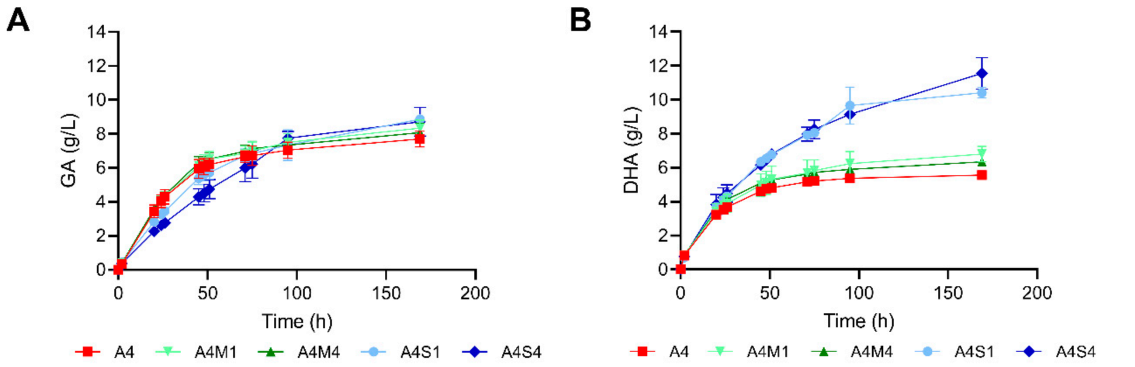

Figure S2. Glycerol bioconversions with Gfr immobilized preparations. A) Kinetics of GA production with A4 (red squares), A4M1 (light green inverted triangles), A4M4 (dark green triangles), A4S1 (light blue circles), and A4S4 (dark blue rhombuses). B) Kinetics of DHA production with A4 (red squares), A4M1 (light green inverted triangles), A4M4 (dark green triangles), A4S1 (light blue circles), and A4S4 (dark blue rhombuses).

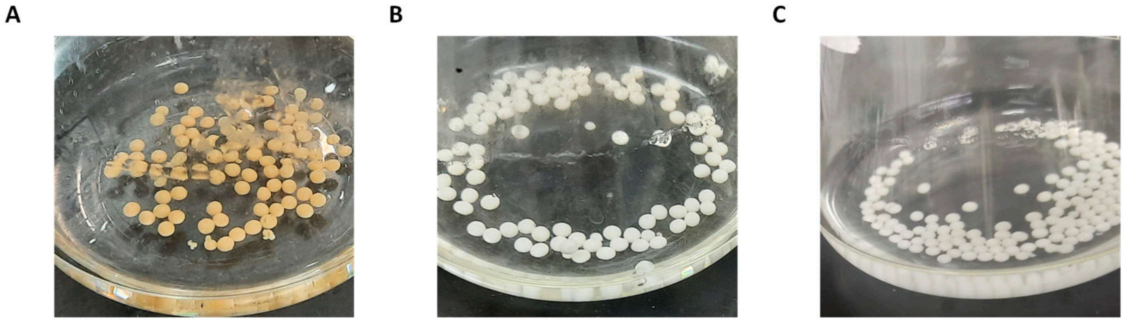

Figure S3. Evaluation of color change after 24 hours in A4S4 preparations. A) Gfr immobilized in A4S4 in presence of 200 g/L glycerol. B) Gfr immobilized in A4S4 in water without glycerol. C) bacteria-free A4S4 preparations in presence of 200 g/L glycerol.

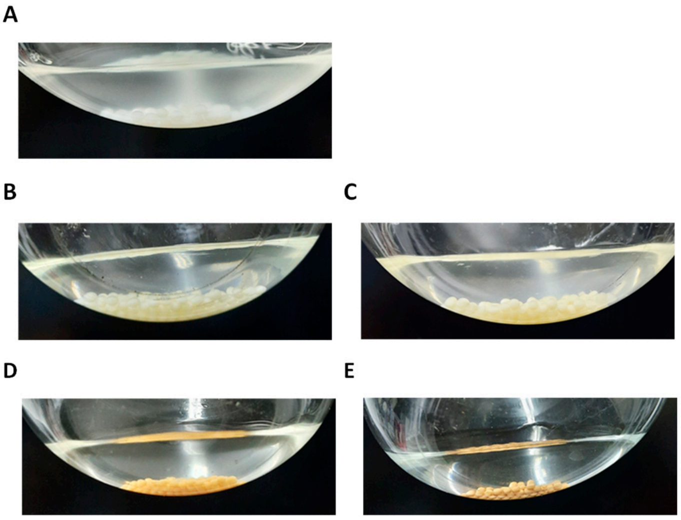

Figure S4 Reaction supernatants after 170 hours of glycerol conversion reactions carried out by different Gfr immobilized preparations. A) A4. B) A4M1. C) A4M4. D) A4S1. E) A4S4.

Table S1 Gfr immobilization in natural polymers.

Immobilized preparation	Bead quantity	Initial diameter (mm)	Final diameter (mm)[a]
AR3	49 ± 8	4.7 ± 1.3	4.0 ± 1.2
AE3	51 ± 6	5.1 ± 0.8	5.0 ± 0.5
A4	156 ± 8	3.2 ± 0.3	2.9 ± 0.2

[a] After 20 hours in reaction medium

Table S2. Diameter evaluation of A4 and A4S4 preparations before and after 8 consecutive uses.

Immobilized preparation	Initial diameter (mm)	Final diameter (mm)
A4	3.8 ± 0.3	nd[a]
A4S4	3.9 ± 0.3	2.6 ± 0.3

[a] Diameter was not determined as the beads were all damaged and cut in halves.
